# Missense Gamma-Aminobutyric Acid Receptor Polymorphisms Are Associated with Reaction Time, Motor Time, and Ethanol Effects *in Vivo*

**DOI:** 10.3389/fncel.2018.00010

**Published:** 2018-01-31

**Authors:** Elena García-Martín, María I. Ramos, José A. Cornejo-García, Segismundo Galván, James R. Perkins, Laura Rodríguez-Santos, Hortensia Alonso-Navarro, Félix J. Jiménez-Jiménez, José A. G. Agúndez

**Affiliations:** ^1^Department of Pharmacology, Universidad de Extremadura, Cáceres, Spain; ^2^ARADyAL Network, Instituto de Salud Carlos III, Madrid, Spain; ^3^Department of Psychiatry, Universidad de Extremadura, Badajoz, Spain; ^4^Research Laboratory, Instituto de Investigación Biomédica de Málaga, Regional University Hospital of Malaga, UMA, Malaga, Spain; ^5^Section of Neurology, Hospital Universitario del Sureste, Madrid, Spain

**Keywords:** GABA-A receptors, ethanol, polymorphism, single nucleotide, reaction time, movement time

## Abstract

**Background:** The Gamma-aminobutyric acid type A receptor (GABA-A receptor) is affected by ethanol concentrations equivalent to those reached during social drinking. At these concentrations, ethanol usually causes impairment in reaction and motor times in most, but not all, individuals.

**Objectives:** To study the effect of GABA-A receptor variability in motor and reaction times, and the effect of low ethanol doses.

**Methods:** Two hundred and fifty healthy subjects received one single dose of 0.5 g/Kg ethanol *per os*. Reaction and motor times were determined before ethanol challenge (basal), and when participants reached peak ethanol concentrations. We analyzed all common missense polymorphisms described in the 19 genes coding for the GABA-A receptor subunits by using TaqMan probes.

**Results:** The *GABRA6* rs4454083 T/C polymorphisms were related to motor times, with individuals carrying the C/C genotype having faster motor times, both, at basal and at peak ethanol concentrations. The *GABRA4* rs2229940 T/T genotype was associated to faster reaction times and with lower ethanol effects, determined as the difference between basal reaction time and reaction time at peak concentrations. All these associations remained significant after correction for multiple comparisons. No significant associations were observed for the common missense SNPs *GABRB3* rs12910925, *GABRG2* rs211035, *GABRE* rs1139916, *GABRP* rs1063310, *GABRQ* rs3810651, *GABRR1* rs12200969 or rs1186902, *GABRR2* rs282129, and *GABRR3* rs832032.

**Conclusions:** This study provides novel information supporting a role of missense GABA-A receptor polymorphisms in reaction time, motor time and effects of low ethanol doses *in vivo*.

## Introduction

The Gamma-aminobutyric acid type A receptor (GABA-A receptor) is a ligand-gated ion channel that plays a key role in the inhibition of neurotransmission because, when activated by GABA, it causes neuronal hyperpolarization. The activation of the GABA-A receptor by ethanol has long been well-known (Hunt, 1983; Kulonen, 1983; Leidenheimer and Harris, 1992; Aguayo et al., 2002; Chester and Cunningham, 2002; Lobo and Harris, 2008; Kumar et al., 2009b; Zuo et al., 2017), and it is related to the motor effects of ethanol. Independent studies have shown that ethanol exposure modulates neuronal function by potentiating GABA-A effects, whereas ethanol effects are blocked by GABA-A antagonists such as bicuculline, Ro15-1788, or picrotoxin (Mehta and Ticku, 1988; Ticku, 1989, 1990; Allan et al., 1991; Miczek et al., 1997). Moreover, GABA-A antagonists seem to reduce ethanol self-administration (Chester and Cunningham, 2002; Holtyn et al., 2017) and GABA system has been proposed as a therapeutic target for alcoholism therapy (Caputo and Bernardi, 2010; Liang and Olsen, 2014; Long et al., 2017; Prisciandaro et al., 2017). In addition, chronic ethanol exposure modifies the expression of some GABA-A receptors subunits (Wallner et al., 2014; Wang et al., 2014; Bekdash and Harrison, 2015; Ortiz et al., 2015; Bohnsack et al., 2016, 2017; Carlson et al., 2016), thus suggesting the involvement of GABA-A in ethanol tolerance and dependence (Mhatre and Ticku, 1993; Grobin et al., 2000; Davies, 2003; Kumar et al., 2004; Blednov et al., 2017; Caputo et al., 2017; Chandler et al., 2017; Lieberman et al., 2017; Lindemeyer et al., 2017; McCabe et al., 2017). Other effects induced by chronic ethanol exposure on GABA-A are changes in receptor function, phosphorylation, synaptic localization and changes in the receptor subunit composition (Kumar et al., 2004; Follesa et al., 2006; Weiner and Valenzuela, 2006; Enoch, 2008).

Human GABA-A receptors are pentamers, composed of a combination of 19 different subunits, namely, six GABRα (GABRα1 to GABRα6), three GABRβ (GABRβ1 to GABRβ3), three GABRγ (GABRγ1 to GABRγ3), one each of GABRδ, GABRε, GABRπ and GABRθ (GABRQ) and three GABRρ (GABRρ1 to GABRρ3). The major isoform consists of α1, β2, and γ2 subunits, but many other combinations exist, and it is widely accepted that the subunit combination plays a key role in the function of these receptors (Olsen and Sieghart, 2008, 2009; Sigel and Steinmann, 2012; Martenson et al., 2017). Some subunits and some combinations seem to be more sensitive to ethanol effects than others. It has been shown that highest sensitivity to ethanol is observed with the combination GABRα4 + GABRβ3 + GABRδ and GABRα6 + GABRβ3 + GABRδ (Hanchar et al., 2004). Receptors with such particular subunit combinations are affected by low ethanol concentrations (Wallner et al., 2006; Lovinger and Homanics, 2007), although these findings are not free of controversy (Borghese and Harris, 2007; Botta et al., 2007; Jia et al., 2007; Korpi et al., 2007; Mody et al., 2007; Olsen et al., 2007; Santhakumar et al., 2007).

The response to ethanol *in vivo* is very variable and many factors, besides variability in ethanol metabolism, modify ethanol response. Sex differences in the sensitivity of GABA-A receptors have been shown (Finn et al., 2010) but, interestingly, it has been reported that most of the interindividual variability in the response to ethanol depends on inheritance (Matsushita and Higuchi, 2014). This suggest that genetic variations may play a relevant role in ethanol effects.

In animal models, alteration in the response to ethanol has been related to amino-acid changes in some GABA-A receptor subunits (Loh and Ball, 2000), and it has been suggested that inter- individual variability in the motor impairment caused by low doses of ethanol are related to genetic variability in genes coding for GABA-A receptor subunits (Korpi, 1994; Wallner et al., 2006). For instance, in animal models, a mutation in the GABRα6 subunit has been associated with motor impairment and ethanol sensitivity (Wallner et al., 2006). Information about the putative role of GABA-A genetic variations in ethanol effects in humans, however, is scarce.

The 19 human genes coding for GABA-A receptor subunits contain several common missense polymorphisms (Table 1). Some of the amino acid substitutions have been classified as deleterious according to Sorting Intolerant From Tolerant (SIFT) (Kumar et al., 2009a) and/or Polymorphism Phenotyping v2 (PolyPhen-2) prediction (Adzhubei et al., 2010). In a previous study we observed that a relevant percentage of the population, when exposed to low ethanol doses, did not diminish their reaction time and motor time, but rather were not significantly affected or it even improved their performance (Martinez et al., 2010). The reason for this dimorphism in the response to ethanol is unknown, although we demonstrated that it is not related to ethanol pharmacokinetics (Martinez et al., 2010). This raises the question of whether pharmacodynamic mechanisms, or genetic predisposition, may be involved in such inter-individual variability to ethanol response. In this study, we investigated whether common missense *GABR* polymorphisms are modifiers of response to low doses of ethanol *in vivo*, with the focus on effects on motor and reaction times.

**Table 1:** Common stop and missense SNPs in *GABR* genes and allele frequencies according the 1,000 genomes database.

**Gene**	**Chromosomal location**	**Missense SNPs with minor allele frequency over 0.1: Random SNP Id**.	**Overall frequency**	**Frequency in europeans**	**Amino acid substitution**	**Genotyping assay ID**
*GABRA1*	Chromosome 5: 161,274,197–161,326,975	None	–	–	–	–
*GABRA2*	Chromosome 4: 46,250,444–46,477,247	None	–	–	–	–
*GABRA3*	Chromosome X: 151,334,706–151,619,830	None	–	–	–	–
*GABRA4*	Chromosome 4: 46,920,917–46,996,424	rs2229940	0.301	0.370	M26L	C__22274760_10
*GABRA5*	Chromosome 15: 27,111,510–27,194,354	None	–	–	–	–
*GABRA6*	Chromosome 5: 160,974,069–161,129,599	rs4454083	0.262	0.300	L13P	C____220485_10
*GABRB1*	Chromosome 4: 46,995,740–47,428,461	None	–	–	–	–
*GABRB2*	Chromosome 5: 160,715,436–160,976,050	None	–	–	–	–
*GABRB3*	Chromosome 15: 26,788,693–27,184,686	rs17647384	0.338	0.460	R23C	–^a^
*GABRB3*	Chromosome 15: 26,788,693–27,184,686	rs12910925	0.338	0.460	R50W	C__11753146_10
*GABRG1*	Chromosome 4: 46,037,786–46,126,098	None	–	–	–	–
*GABRG2*	Chromosome 5: 161,494,546–161,582,542	rs211035	0.179	0.200	I215V	C__26181781_10
*GABRG3*	Chromosome 15: 27,216,429–27,778,373	None	–	–	–	–
*GABRD*	Chromosome 1: 1,950,780–1,962,192	None	–	–	–	–
*GABRE*	Chromosome X: 151,121,596–151,143,152	rs1139916	0.294	0.300	S102A	C__15868735_20
*GABRP*	Chromosome 5: 170,190,354–170,241,051	rs1063310	0.224	0.260	F391L	C___8953491_20
*GABRQ*	Chromosome X: 151,806,637–151,825,999	rs3810651	0.435	0.440	I478F	C__27492635_10
*GABRR1*	Chromosome 6: 89,887,220–89,940,997	rs12200969	0.306	0.350	M26V	C__11420642_10
*GABRR1*	Chromosome 6: 89,887,220–89,940,997	rs1186902	0.231	0.260	H27R	C___8945590_20
*GABRR2*	Chromosome 6: 89,966,927–90,025,018	rs282129	0.252	0.250	T430M	C___2332356_20
*GABRR3*	Chromosome 3: 97,705,517–97,754,148	rs832032	0.173	0.190	Y205Stop	C___8751472_10

a *This SNP was not tested because it is at complete linkage with another common SNP (rs12910925) located in the same gene*.

## Methods

This study was performed on a group of individuals involved in a previous study on the effect of variability of genes coding for ethanol-metabolizing enzymes in ethanol pharmacokinetics and effects (Martinez et al., 2010). The study group consisted of 250 healthy individuals (66 men and 184 women), with a mean age equal to 22.5 ± 6.2 years. All were Spanish Caucasian individuals recruited at the Medical School of the University of Extremadura (Badajoz, Spain) among students and staff. The inclusion criteria included absence of ethanol consumption, absence of consumption of drugs with known effects in the nervous system, as well as illicit drugs 7 days before the study. We recorded data on age, sex, smoking, and drinking habits. Signed informed consent was obtained for all participants. The protocol of the study was approved by the Ethics Committee of the University Hospital Infanta Cristina (Badajoz, Spain).

### Ethanol challenge and determination of ethanol effects

The design of ethanol challenge and the pharmacokinetic analysis is described elsewhere (Martinez et al., 2010). In brief, participants started the challenge at the same hour, after overnight fasting. Participants received 0.5 g/Kg ethanol *per os* as liquor containing 17% ethanol. In order to determine when the peak concentration was reached, breath samples were measured every 15 min by using a breath analyzer Alcotest-7710 (Dräger Safety, Spain). Pharmacokinetic determinations were carried out until ethanol levels reached values lower than 0.02 g/L in air, or during 5 h.

After training, participants carried out tests designed to determine reaction time and motor time by using the Vienna Test System 5.20 (Dr. G. Schuhfried GMBH 2003, Austria). Each participant carried out one test just before the ethanol challenge and then one test every 30 min. Each time, 16 measurements of reaction time and motor time were carried out, and the average values for these determinations were used for further analyses. Test takers had their finger on a gold button, moving it to press a black button when they perceived a determined visual stimuli. Reaction time is defined as the time lapsed between displaying the stimulus and removing the finger from the gold button, and motor time is defined as the time lapsed between removing the finger from the gold button until the black button is pressed. To assess ethanol effect on the reaction times and in the motor times, we compared these parameters at basal conditions (the average of the results before ethanol administration and after ethanol concentration in breath were lower than 0.05 g/L) with those obtained at peak ethanol concentration.

### Genotyping

Genomic DNA was obtained from venous blood as described elsewhere. The selection of the non-synonymous *GABRR* polymorphisms analyzed was made according to the expected allele frequencies in the population study previously reported in public databases such as the public 1,000 genomes database^1^ The study was designed to investigate all non-synonymous and/or nonsense SNPs with minor allele frequencies (MAF) higher than 0.1 for individuals of European descent, for the 19 GABA-A receptor genes described in Table 1. SNPs with lower MAF were not included in the study because the statistical power would be insufficient to obtain conclusive evidence. According to the 1,000 genomes database, 12 SNPs fulfilled the selection criteria. However, two *GABRB3* SNPs, namely rs12910925 and rs17647384, are at complete linkage, and therefore only the former was included in the study. Genotyping was carried out in triplicate using SNP TaqMan assays (Life Technologies S.A., Alcobendas, Madrid, Spain). Further details on Assay ID are provided in Table 1.

### Statistical analyses

Statistical analyses were carried out by using the IBM SPSS Statistical Package Version 22 (International Business Machines Corporation, Madrid, Spain). Intergroup comparison values were calculated by using the *T*-test when appropriate. After normality test, multiple comparison of means was carried out by using the Kruskal–Wallis test for independent samples, because most parameters did not follow a normal distribution. Multiple testing correction was carried out by means of the False Discovery Rate (FDR) procedure (Benjamini and Hochberg, 1995). Haplotypes were calculated as described elsewhere (Mateos-Munoz et al., 2016). The Hardy–Weinberg equilibrium was analyzed through the DeFinetti procedure (http://ihg.gsf.de/cgi-bin/hw/hwa1.pl).

## Results

*GABRA* genotyping results stratified by sex are summarized in Table 2. The genotypes and allele frequencies observed for the SNP analyzed are consistent with those reported elsewhere for healthy Spanish individuals (Garcia-Martin et al., 2011a,b, 2017) and with those reported in public databases^2^ All SNPs were at Hardy-Weinberg's equilibrium with the exception of the SNPs *GABRE* rs1139916 and *GABRQ* rs3810651, which correspond to genes located in the X chromosome (as shown in Table 1). These two SNPs were at Hardy-Weinberg's equilibrium in women (Pearson's goodness-of-fit chi-square *P* = 0.361 and *P* = 0.707, respectively), but not in men, as is to be expected in genes located on chromosome X.

**Table 2:** *GABR* genotypes and allele frequencies distributed by sex.

**Genotypes**	**Women (No; 95% CI)**	**Men (No; 95% CI)**
*GABRA4* rs2229940 G/G	73 (38.6; 31.7 to 45.6)	26 (42.6; 30.2 to 55.0)
*GABRA4* rs2229940 G/T	87 (46.0; 38.9 to 53.1)	23 (37.7; 25.5 to 49.9)
*GABRA4* rs2229940 T/T	29 (15.3; 10.2 to 20.5)	12 (19.7; 9.7 to 29.6)
*GABRA6* rs4454083 T/T	99 (52.4; 45.3 to 59.5)	29 (47.5; 35.0 to 60.1)
*GABRA6* rs4454083 T/C	66 (34.9; 28.1 to 41.7)	26 (42.6; 30.2 to 55.0)
*GABRA6* rs4454083 C/C	24 (12.7; 8.0 to 17.4)	6 (9.8; 2.4 to 17.3)
*GABRB3* rs12910925 C/C	54 (28.6; 22.1 to 35.0)	17 (27.9; 16.6 to 39.1)
*GABRB3* rs12910925 C/T	96 (50.8; 43.7 to 57.9)	29 (47.5; 35.0 to 60.1)
*GABRB3* rs12910925 T/T	39 (20.6; 14.9 to 26.4)	15 (24.6; 13.8 to 35.4)
*GABRG2* rs211035 G/G	133 (70.4; 63.9 to 76.9)	40 (65.6; 53.7 to 77.5)
*GABRG2* rs211035 G/A	54 (28.6; 22.1 to 35.0)	21 (34.4; 22.5 to 46.3)
*GABRG2* rs211035 A/A	2 (1.1; −0.4 to 2.5)	0 (0.0; 0.0 to 0.0)
*GABRE* rs1139916 C/C	76 (40.2; 33.2 to 47.2)	43 (70.5; 59.0 to 81.9)
*GABRE* rs1139916 C/A	83 (43.9; 36.8 to 51.0)	–
*GABRE* rs1139916 A/A	30 (15.9; 10.7 to 21.1)	18 (29.5; 18.1 to 41.0)
*GABRP* rs1063310 C/C	112 (59.3; 52.3 to 66.3)	38 (62.3; 50.1 to 74.5)
*GABRP* rs1063310 C/A	67 (35.4; 28.6 to 42.3)	21 (34.4; 22.5 to 46.3)
*GABRP* rs1063310 A/A	10 (5.3; 2.1 to 8.5)	2 (3.3; −1.2 to 7.7)
*GABRQ* rs3810651 T/T	70 (37.0; 30.2 to 43.9)	35 (57.4; 45.0 to 69.8)
*GABRQ* rs3810651 T/A	88 (46.6; 39.4 to 53.7)	–
*GABRQ* rs3810651 A/A	31 (16.4; 11.1 to 21.7)	26 (42.6; 30.2 to 55.0)
*GABRR1* rs12200969 T/T	99 (52.4; 45.3 to 59.5)	35 (57.4; 45.0 to 69.8)
*GABRR1* rs12200969 T/C	69 (36.5; 29.6 to 43.4)	21 (34.4; 22.5 to 46.3)
*GABRR1* rs12200969 C/C	21 (11.1; 6.6 to 15.6)	5 (8.2; 1.3 to 15.1)
*GABRR1* rs1186902 T/T	172 (91.0; 86.9 to 95.1)	54 (88.5; 80.5 to 96.5)
*GABRR1* rs1186902 T/C	17 (9.0; 4.9 to 13.1)	7 (11.5; 3.5 to 19.5)
*GABRR1* rs1186902 C/C	0 (0.0; 0.0 to 0.0)	0 (0.0; 0.0 to 0.0)
*GABRR2* rs282129 G/G	126 (66.7; 59.9 to 73.4)	39 (63.9; 51.9 to 76.0)
*GABRR2* rs282129 G/A	55 (29.1; 22.6 to 35.6)	21 (34.4; 22.5 to 46.3)
*GABRR2* rs282129 A/A	8 (4.2; 1.4 to 7.1)	1 (1.6; −1.5 to 4.8)
*GABRR3* rs832032 A/A	115 (60.8; 53.9 to 67.8)	39 (63.9; 51.9 to 76.0)
*GABRR3* rs832032 A/T	66 (34.9; 28.1 to 41.7)	20 (32.8; 21.0 to 44.6)
*GABRR3* rs832032 T/T	8 (4.2; 1.4 to 7.1)	2 (3.3; −1.2 to 7.7)
**Alleles**	**Women (No; 95% CI)**	**Men (No; 95% CI)**
*GABRA4* rs2229940 G	233 (61.6; 56.7 to 66.5)	75 (61.5; 52.8 to 70.1)
*GABRA4* rs2229940 T	145 (38.4; 33.5 to 43.3)	47 (38.5; 29.9 to 47.2)
*GABRA6* rs4454083 T	264 (69.8; 65.2 to 74.5)	84 (68.9; 60.6 to 77.1)
*GABRA6* rs4454083 C	114 (30.2; 25.5 to 34.8)	38 (31.1; 22.9 to 39.4)
*GABRB3* rs12910925 C	204 (54.0; 48.9 to 59.0)	63 (51.6; 42.8 to 60.5)
*GABRB3* rs12910925 T	174 (46.0; 41.0 to 51.1)	59 (48.4; 39.5 to 57.2)
*GABRG2 r*s211035 G	320 (84.7; 81.0 to 88.3)	101 (82.8; 76.1 to 89.5)
*GABRG2* rs211035 A	58 (15.3; 11.7 to 19.0)	21 (17.2; 10.5 to 23.9)
*GABRE* rs1139916 C	235 (62.2; 57.3 to 67.1)	43 (70.5; 59.0 to 81.9)
*GABRE* rs1139916 A	143 (37.8; 32.9 to 42.7)	18 (29.5; 18.1 to 41.0)
*GABRP* rs1063310 C	291 (77.0; 72.7 to 81.2)	97 (79.5; 72.3 to 86.7)
*GABRP* rs1063310 A	87 (23.0; 18.8 to 27.3)	25 (20.5; 13.3 to 27.7)
*GABRQ* rs3810651 T	228 (60.3; 55.4 to 65.2)	35 (57.4; 45.0 to 69.8)
*GABRQ* rs3810651 A	150 (39.7; 34.8 to 44.6)	26 (42.6; 30.2 to 55.0)
*GABRR1* rs12200969 T	267 (70.6; 66.0 to 75.2)	91 (74.6; 66.9 to 82.3)
*GABRR1* rs12200969 C	111 (29.4; 24.8 to 34.0)	31 (25.4; 17.7 to 33.1)
*GABRR1* rs1186902 T	361 (95.5; 93.4 to 97.6)	115 (94.3; 90.1 to 98.4)
*GABRR1* rs1186902 C	17 (4.5; 2.4 to 6.6)	7 (5.7; 1.6 to 9.9)
*GABRR2* rs282129 G	307 (81.2; 77.3 to 85.2)	99 (81.1; 74.2 to 88.1)
*GABRR2* rs282129 A	71 (18.8; 14.8 to 22.7)	23 (18.9; 11.9 to 25.8)
*GABRR3* rs832032 A	296 (78.3; 74.2 to 82.5)	98 (80.3; 73.3 to 87.4)
*GABRR3* rs832032 T	82 (21.7; 17.5 to 25.8)	24 (19.7; 12.6 to 26.7)

The ethanol challenge was designed to obtain low peak concentrations, comparable to the ethanol concentrations reached during social drinking, that is, between two and four drinks depending on body weight (one drink being equal to 14 g of pure ethanol). In the population studied, peak concentrations in breath ranged from 0.19 to 0.65 g/L (average 0.42 ± 0.09 g/L) and peak concentrations were reached in the range 15 to 90 min after ethanol ingestion (mean 37.0 ± 16.8 min). We described previously in this study group that no statistically significant associations of reaction or motor times with peak concentration or drinking habits were observed, but average motor values, both, basal, and peak motor times, were shorter in men than in women (Martinez et al., 2010), and the statistical association remained significant after FDR correction for multiple testing (Pc = 0.030 for basal motor time and Pc = 0.012 for peak motor time). The finding that motor times are faster in men than in women was reported previously in another study involving different participants (Jimenez-Jimenez et al., 2011).

In order to test the putative effect of the *GABR* genotypes analyzed on reaction and motor times, we stratified the population according to the 11 SNPs studied. Table 3 shows the distribution of the reaction times according to *GABR* genotypes. Basal reaction time, peak reaction time, and the difference (peak time minus basal time) were shorter among individuals carrying the *GABRA4* rs2229940 T/T genotype, as compared to individuals carrying at least one non-mutated gene. The K–W test remains significant for peak reaction time and the difference (peak time minus basal time), even after correction for multiple comparison. In addition, the *T*-test revealed significant differences when comparing individuals with the rs2229940 T/T genotype with individuals carrying at least one non-mutated gene: *P* = 0.031, P < 0.001 and *P* = 0.001 for basal, peak and the difference in reaction time, respectively. This implies that individuals with GABRA4 receptors with a leucine in position 26 react faster, and are less sensitive to ethanol, as compared with individuals that have a methionine instead. The percentage of variation of the basal functional response was 14.97 ± 15.23 for individuals with the G/G genotype, 14.07 ± 13.04 for individuals with the G/T genotype, and 6.51 ± 14.34 for individuals with the T/T genotype (K–W test *P* = 0.005). Another significant effect was observed in the difference in the reaction time for individuals with *GABRR1* rs12200969 C/C genotype (crude *P* = 0.017; Table 3). The percentage of variation of the basal functional response was 15.60 ± 14.91 for individuals with the T/T genotype, 10.49 ± 13.14 for individuals with the T/C genotype, and 10.92 ± 14.20 for individuals with the C/C genotype (K–W test *P* = 0.017). However, in this case the K–W test did not remain significant after correction for multiple comparisons, and nor did the *T*-test when individuals were compared with the rs12200969 C/C genotype with individuals carrying at least one non-mutated gene: *P* = 0.068.

**Table 3:** Distribution of reaction times according *GABR* genotypes.

	**Basal reaction time (Ms) ± *SD***	**Reaction time at ethanol Peak (Msec) ± *SD***	**Difference (Ms) ± *SD***
*GABRA4* rs2229940 G/T	G/G: 452.60 ± 88.63 G/T: 484.66 ± 89.46 T/T: 444.99 ± 58.39 K–W test, *P* = 0.007; Pc = 0.077	G/G: 515.12 ± 93.31 G/T: 548.99 ± 98.63 T/T: 471.32 ± 71.91 K–W test, *P* = 0.001; Pc = 0.011	G/G: 62.51 ± 63.67 G/T: 64.33 ± 55.77 T/T: 26.32 ± 62.21 K–W test, *P* = 0.002; Pc = 0.022
*GABRA6* rs4454083 T/C	T/T: 465.11 ± 88.70 T/C: 468.15 ± 83.33 C/C: 441.25 ± 64.09 K–W test, *P* = 0.484; Pc = 0.591	T/T: 513.12 ± 95.32 T/C: 533.43 ± 101.35 C/C: 509.76 ± 65.54 K–W test, *P* = 0.417; Pc = 0.655	T/T: 48.00 ± 63.13 T/C: 65.28 ± 62.59 C/C: 68.51 ± 53.51 K–W test, *P* = 0.081; Pc = 0.223
*GABRB3* rs12910925 C/T	C/C: 463.55 ± 76.01 C/T: 464.92 ± 89.30 T/T: 466.97 ± 77.12 K–W test, *P* = 0.895; Pc = 0.967	C/C: 512.87 ± 86.59 C/T: 523.52 ± 97.35 T/T: 523.51 ± 99.92 K–W test, *P* = 0.832; Pc = 0.984	C/C: 49.32 ± 55.11 C/T: 58.61 ± 59.04 T/T: 56.55 ± 75.27 K–W test, *P* = 0.553; Pc = 0.676
*GABRG2* rs211035 G/A	G/G: 460.17 ± 85.86 G/A: 469.96 ± 78.56 A/A: 548.75 ± 61.16 K–W test, *P* = 0.227; Pc = 0.528	G/G: 516.81 ± 92.47 G/A: 529.31 ± 100.17 A/A: 503.50 ± 161.93 K–W test, *P* = 0.897; Pc = 0.984	G/G: 56.63 ± 58.82 G/A: 59.34 ± 66.65 A/A: 45.25 ± 100.76 K–W test, *P* = 0.210; Pc = 0.400
*GABRE* rs1139916 C/A	C/C: 460.48 ± 95.58 C/A: 467.05 ± 78.86 A/A: 477.62 ± 79.80 K–W test, *P* = 0.355; Pc = 0.542	C/C: 523.94 ± 103.39 C/A: 525.22 ± 100.02 A/A: 520.64 ± 79.80 K–W test, *P* = 0.984; Pc = 0.984	C/C: 63.46 ± 66.58 C/A: 58.17 ± 54.98 A/A: 43.02 ± 55.53 K–W test, *P* = 0.218; Pc = 0.400
*GABRP* rs1063310 C/A	C/C: 473.42 ± 83.54 C/A: 454.85 ± 81.03 A/A: 466.97 ± 95.61 K–W test, *P* = 0.279; Pc = 0.528	C/C: 528.79 ± 98.44 C/A: 507.94 ± 88.97 A/A: 549.27 ± 93.39 K–W test, *P* = 0.206; Pc = 0.567	C/C: 55.36 ± 66.72 C/A: 53.09 ± 50.86 A/A: 82.30 ± 73.66 K–W test, *P* = 0.509; Pc = 0.676
*GABRQ* rs3810651 T/A	T/T: 464.03 ± 83.22 T/A: 464.20 ± 85.38 A/A: 473.42 ± 97.88 K–W test, *P* = 0.967; Pc = 0.967	T/T: 519.73 ± 95.53 T/A: 522.95 ± 93.95 A/A: 536.18 ± 105.68 K–W test, *P* = 0.823; Pc = 0.984	T/T: 55.70 ± 59.80 T/A: 58.75 ± 58.34 A/A: 62.76 ± 67.87 K–W test, *P* = 0.673; Pc = 0.740
*GABRR1* rs12200969 T/C	T/T: 462.98 ± 84.27 T/C: 460.58 ± 82.61 C/C: 502.80 ± 105.46 K–W test, *P* = 0.065; Pc = 0.358	T/T: 530.10 ± 89.71 T/C: 506.08 ± 93.36 C/C: 555.30 ± 128.96 K–W test, *P* = 0.092; Pc = 0.337	T/T: 67.12 ± 61.10 T/C: 45.50 ± 59.04 C/C: 52.50 ± 68.94 K–W test, *P* = 0.017; Pc = 0.094
*GABRR1* rs1186902 T/C	T/T: 466.26 ± 84.26 T/C: 449.31 ± 73.92 C/C: – K–W test, *P* = 0.288; Pc = 0.528	T/T: 522.82 ± 91.97 T/C: 516.29 ± 108.69 C/C: – K–W test, *P* = 0.337; Pc = 0.617	T/T: 56.56 ± 57.96 T/C: 66.97 ± 91.77 C/C: – K–W test, *P* = 0.804; Pc = 0.804
*GABRR2* rs282129 G/A	G/G: 466.32 ± 91.05 G/A: 457.10 ± 69.47 A/A: 501.36 ± 101.12 K–W test, *P* = 0.394; Pc = 0.542	G/G: 529.01 ± 100.32 G/A: 506.82 ± 83.62 A/A: 541.37 ± 95.93 K–W test, *P* = 0.259; Pc = 0.570	G/G: 62.69 ± 62.44 G/A: 49.72 ± 53.77 A/A: 40.01 ± 35.49 K–W test, *P* = 0.080; Pc = 0.223
*GABRR3* rs832032 A/T	A/A: 457.68 ± 81.01 A/T: 474.20 ± 94.08 T/T: 504.58 ± 105.39 K–W test, *P* = 0.254; Pc = 0.528	A/A: 510.15 ± 92.12 A/T: 542.26 ± 99.86 T/T: 542.50 ± 119.18 K–W test, *P* = 0.064; Pc = 0.337	A/A: 52.47 ± 51.43 A/T: 68.05 ± 71.79 T/T: 37.91 ± 73.90 K–W test, *P* = 0.303, Pc = 0.476

*Kruskal–Wallis Test (K–W) was used because none of the reaction times, nor the differences, followed a normal distribution. Crude P-values are adjusted by sex. Pc-values correspond to the corrected P values calculated according to the FDR procedure*.

Table 4 shows the distribution of the motor times according to *GABR* genotypes. Basal motor time and peak motor time were shorter among individuals carrying the *GABRA6* rs4454083 C/C genotype, as compared with individuals carrying at least one non-mutated gene. The K–W test remains significant for both motor times after correction for multiple comparisons. In addition, the *T*-test reveals very significant differences when individuals with the rs2229940 T/T genotype were compared, with individuals carrying at least one non-mutated gene: *P* < 0.001 and *P* = 0.001 for basal and peak motor time, respectively. This implies that individuals with GABRA6 receptors with a proline in the position 13 instead of leucine have faster motor times. In contrast, no statistically significant effects related to ethanol (that is, the difference between basal motor time minus peak motor time) were observed. The percentage of variation of the basal functional response was 8.38 ± 17.15 for individuals with the T/T genotype, 6.84 ± 17.94 for individuals with the T/C genotype, and 8.99 ± 23.56 for individuals with the C/C genotype (K–W test *P* = 0.746). No other associations with motor times were identified when other *GABR* genotypes were analyzed.

**Table 4:** Distribution of motor times according *GABR* genotypes.

	**Basal motor time (Ms) ± *SD***	**Motor time at ethanol Peak (Ms) ± *SD***	**Difference (Ms) ± *SD***
*GABRA4* rs2229940 G/T	G/G: 172.13 ± 59.74 G/T: 164.40 ± 60.13 T/T: 158.91 ± 43.80 K–W test, *P* = 0.470; Pc = 0.932	G/G: 185.17 ± 60.64 G/T: 173.12 ± 60.30 T/T: 173.17 ± 43.80 K–W test, *P* = 0.302; Pc = 0.977	G/G: 13.04 ± 31.79 G/T: 8.72 ± 31.73 T/T: 14.26 ± 25.37 K–W test, *P* = 0.672; Pc = 0.905
*GABRA6* rs4454083 T/C	T/T: 169.52 ± 50.29 T/C: 177.92 ± 67.37 C/C: 135.66 ± 36.81 K–W test, *P* = 0.003; Pc = 0.033	T/T: 181.70 ± 54.21 T/C: 186.92 ± 66.22 C/C: 146.65 ± 48.99 K–W test, *P* = 0.003; Pc = 0.033	T/T: 12.18 ± 31.66 T/C: 9.00 ± 32.36 C/C: 10.99 ± 28.80 K–W test, *P* = 0.691; Pc = 0.905
*GABRB3* rs12910925 C/T	C/C: 165.12 ± 53.17 C/T: 171.35 ± 63.24 T/T: 161.88 ± 50.04 K–W test, *P* = 0.893; Pc = 0.932	C/C: 177.55 ± 50.89 C/T: 182.42 ± 65.62 T/T: 174.86 ± 54.69 K–W test, *P* = 0.894; Pc = 0.977	C/C: 12.44 ± 34.94 C/T: 11.08 ± 33.44 T/T: 12.99 ± 21.68 K–W test, *P* = 0.797; Pc = 0.905
*GABRG2* rs211035 G/A	G/G: 169.11 ± 60.74 G/A: 164.07 ± 49.55 A/A: 199.11 ± 70.13 K–W test, *P* = 0.364; Pc = 0.932	G/G: 177.84 ± 58.73 G/A: 181.62 ± 61.96 A/A: 210.28 ± 86.14 K–W test, *P* = 0.382; Pc = 0.977	G/G: 8.73 ± 33.15 G/A: 17.55 ± 28.20 A/A: 15.33 ± 8.06 K–W test, *P* = 0.273; Pc = 0.905
*GABRR3* rs832032 A/T	A/A: 163.58 ± 55.78 A/T: 171.03 ± 62.07 T/T: 158.27 ± 45.46 K–W test, *P* = 0.910; Pc = 0.932	A/A: 177.02 ± 59.78 A/T: 177.69 ± 56.19 T/T: 173.20 ± 52.08 K–W test, *P* = 0.976; Pc = 0.977	A/A: 13.44 ± 32.53 A/T: 6.66 ± 28.86 T/T: 14.93 ± 15.14 K–W test, *P* = 0.484; Pc = 0.905
*GABRP* rs1063310 C/A	C/C: 168.35 ± 58.56 C/A: 166.23 ± 58.01 A/A: 155.46 ± 45.59 K–W test, *P* = 0.932; Pc = 0.932	C/C: 180.63 ± 62.54 C/A: 176.08 ± 54.86 A/A: 165.20 ± 53.97 K–W test, *P* = 0.900; Pc = 0.977	C/C: 12.28 ± 30.56 C/A: 9.85 ± 35.09 A/A: 9.74 ± 18.46 K–W test, *P* = 0.871; Pc = 0.905
*GABRQ* rs3810651 T/A	T/T: 169.20 ± 62.59 T/A: 165.28 ± 58.32 A/A: 160.29 ± 45.94 K–W test, *P* = 0.900; Pc = 0.932	T/T: 177.12 ± 54.96 T/A: 177.90 ± 65.43 A/A: 174.23 ± 53.61 K–W test, *P* = 0.977; Pc = 0.977	T/T: 7.93 ± 33.41 T/A: 12.63 ± 25.53 A/A: 13.96 ± 33.54 K–W test, *P* = 0.905; Pc = 0.905
*GABRR1* rs12200969 T/C	T/T: 165.61 ± 50.89 T/C: 164.33 ± 62.72 C/C: 177.77 ± 73.74 K–W test, *P* = 0.647; Pc = 0.932	T/T: 176.91 ± 56.48 T/C: 175.23 ± 63.07 C/C: 181.64 ± 54.43 K–W test, *P* = 0.805; Pc = 0.977	T/T: 11.31 ± 26.84 T/C: 10.90 ± 31.21 C/C: 3.86 ± 44.03 K–W test, *P* = 0.547; Pc = 0.905
*GABRR1* rs1186902 T/C	T/T: 167.07 ± 58.23 T/C: 161.16 ± 52.71 C/C: – K–W test, *P* = 0.595; Pc = 0.932	T/T: 177.82 ± 58.83 T/C: 172.95 ± 54.89 C/C: – K–W test, *P* = 0.624; Pc = 0.977	T/T: 10.75 ± 32.02 T/C: 11.79 ± 24.93 C/C: – K–W test, *P* = 0.903; Pc = 0.905
*GABRR2* rs282129 G/A	G/G: 163.90 ± 51.66 G/A: 172.28 ± 70.29 A/A: 170.10 ± 60.50 K–W test, *P* = 0.896; Pc = 0.932	G/G: 174.78 ± 54.03 G/A: 183.73 ± 67.54 A/A: 188.50 ± 89.55 K–W test, *P* = 0.869; Pc = 0.977	G/G: 10.89 ± 30.55 G/A: 11.45 ± 33.47 A/A: 18.40 ± 36.34 K–W test, *P* = 0.899; Pc = 0.905
*GABRE* rs1139916 C/A	C/C: 159.30 ± 47.89 C/A: 176.77 ± 74.11 A/A: 166.65 ± 49.47 K–W test, *P* = 0.371; Pc = 0.932	C/C: 172.58 ± 52.46 C/A: 184.80 ± 73.69 A/A: 175.25 ± 43.84 K–W test, *P* = 0.891; Pc = 0.977	C/C: 13.28 ± 29.15 C/A: 8.03 ± 28.85 A/A: 8.60 ± 37.49 K–W test, *P* = 0.709; Pc = 0.905

*Kruskal–Wallis Test (K–W) was used because none of the reaction times, nor the differences, followed a normal distribution. Crude P-values are adjusted by sex. Pc-values correspond to the corrected P values calculated according to the FDR procedure*.

Table 5 shows the haplotype analysis results for the whole study group. Due to the high frequency of variant GABR alleles, no common haplotypes were observed. The most common haplotype had a frequency of about 6%, and rare haplotypes (with frequencies lower than 1%) accounted for almost 50% of all haplotypes identified. For that reason, and in order to refine the putative genetic associations, the effect of inferred haplotypes on reaction and motor times was studied for the SNPs that displayed any association in Tables 3, 4, the SNPs associated to the most common GABA-A receptor configuration and the SNPs associated to the subunit combinations that are the most sensitive to ethanol. Table 6 shows the association of haplotypes, adjusted by sex, with reaction and motor times. Several statistically significant associations were identified, even after FDR correction for multiple comparisons. However, some haplotypes caused modest changes in reaction or motor times. In addition, some haplotypes that caused larger changes are extremely rare. Table 6 was sorted by type of effect, then statistical significance and then effect intensity. Only two particular effects corresponded unambiguously to specific variant haplotype distributions, as compared to the reference haplotype GTCGT. These are the haplotype GCTGC (corresponding to variant alleles in *GABRA6* and *GABRB3*), which is related to slower basal movement time, and the haplotype TTTAC (corresponding to variant alleles in *GABRA4, GABRB3, GABRG2*, and *GABRR1*), which is related to faster peak movement time. These two haplotypes, however, are extremely rare.

**Table 5:** *GABR* haplotypes in the whole study group.

	**GABRA4 rs2229940**	**GABRA6 rs4454083**	**GABRB3 rs12910925**	**GABRG2 rs211035**	**GABRR3 rs832032**	**GABRP rs1063310**	**GABRQ rs3810651**	**GABRR1 rs12200969**	**GABRR1 rs1186902**	**GABRR2 rs282129**	**GABRE rs1139916**	**Haplotype Frequency**
1	G	T	T	G	C	C	T	T	T	G	A	0.0641
2	G	T	C	G	C	C	A	T	T	G	A	0.0503
3	G	T	C	G	A	C	T	T	T	G	A	0.0406
4	T	T	C	G	C	C	T	T	T	G	A	0.0358
5	T	T	T	G	C	C	T	T	T	G	A	0.0240
6	G	T	T	G	A	C	A	T	T	G	A	0.0224
7	T	T	T	G	A	C	T	T	T	G	A	0.0223
8	G	C	T	G	C	C	A	T	T	G	A	0.0201
9	G	C	C	G	A	C	A	T	T	G	A	0.0197
10	G	T	C	G	C	C	T	T	T	G	A	0.0197
11	G	T	C	G	C	C	T	C	T	G	A	0.0172
12	G	T	C	G	C	A	T	T	T	G	A	0.0159
13	G	C	T	G	C	C	T	C	T	A	A	0.0156
14	G	T	C	G	C	C	A	C	T	G	A	0.0150
15	G	C	C	G	A	C	T	T	T	G	A	0.0145
16	G	T	T	G	C	C	A	T	T	G	A	0.0141
17	T	T	T	G	C	A	A	T	T	G	A	0.0138
18	G	T	C	G	A	C	A	T	T	G	A	0.0138
19	T	C	T	A	C	A	A	T	T	G	A	0.0129
20	G	T	T	G	C	C	T	C	T	G	A	0.0126
21	T	T	C	G	A	C	A	C	T	A	A	0.0118
22	G	T	T	G	C	C	A	T	T	A	A	0.0116
23	T	C	T	G	C	A	T	T	T	G	T	0.0114
Rare haplotypes	–	–	–	–	–	–	–	–	–	–	–	0.4691

**Table 6:** Statistically significant interactions of selected GABR haplotypes with reaction and motor times.

	**GABRA4 rs2229940**	**GABRA6 rs4454083**	**GABRB3 rs12910925**	**GABRG2 rs211035**	**GABRR1 rs12200969**	**Haplotype frequency**	**Difference Ms (95% CI)**	***P*-value**	**Pc**
Reference haplotype	G	T	C	G	T	0.1483	0.00	–	–
Basal RT: Faster	G	T	C	G	C	0.0100	−69.94 (−72.71 to −67.17)	<0.0001	0.0002
Basal RT: Faster	G	C	C	A	T	0.0149	−63.08 (−65.92 to −60.23)	<0.0001	0.0002
Basal RT: Faster	T	T	C	A	T	0.0171	−25.09 (−28.38 to −21.8)	<0.0001	0.0002
Basal RT: Faster	T	C	T	A	T	0.0231	−21.74 (−31.04 to −12.43)	<0.0001	0.0002
Basal RT: Faster	G	T	T	A	T	0.0230	−19.38 (−25.63 to −13.14)	<0.0001	0.0002
Basal RT: Slower	T	T	T	A	T	0.0131	202.36 (200.22 to 204.49)	<0.0001	0.0002
Basal RT: Slower	G	T	C	A	T	0.0156	42.96 (39.25 to 46.66)	<0.0001	0.0002
Peak RT: Faster	T	T	T	G	C	0.0241	−29.39 (−40.4 to −18.37)	<0.0001	0.0004
Peak RT: Faster	T	C	C	A	T	0.0212	−24.59 (−39.19 to −9.99)	0.0009	0.0027
Peak RT: Faster	T	C	T	G	T	0.0267	−28.71 (−46.27 to −11.14)	0.0014	0.0033
Peak RT: Faster	G	C	T	A	T	0.0197	−10.61 (−19.95 to −1.27)	0.0260	0.0494
Peak RT: Slower	T	C	T	A	T	0.0070	249.37 (246.13 to 252.6)	<0.0001	0.0004
Peak RT: Slower	T	C	C	G	C	0.0086	156.56 (153.04 to 160.07)	<0.0001	0.0004
Peak RT: Slower	G	C	T	G	C	0.0230	145.03 (139.91 to 150.15)	<0.0001	0.0004
Peak RT: Slower	T	T	T	A	C	0.0091	121.38 (116.19 to 126.56)	<0.0001	0.0004
Peak RT: Slower	G	T	T	A	T	0.0390	68.55 (28.02 to 109.08)	0.0009	0.0027
Peak RT: Slower	G	C	T	G	T	0.0496	53.65 (14.27 to 93.03)	0.0076	0.0160
Peak RT: Slower	T	T	C	G	C	0.0390	13.18 (0.3 to 26.06)	0.0450	0.0777
Difference RT: Lower	T	T	T	G	C	0.0187	−32.22 (−57.64 to −6.81)	0.0130	0.0867
Difference RT: Larger	T	C	T	A	T	0.0035	103.36 (100.3 to 106.41)	<0.0001	0.0010
Difference RT: Larger	T	C	C	G	C	0.0166	53.79 (45.97 to 61.6)	<0.0001	0.0010
Difference RT: Larger	G	T	T	A	T	0.0371	37.66 (0.74 to 74.57)	0.0460	0.2300
Basal MT: Slower	G	C	T	G	C	0.0070	165.46 (113.85 to 217.06)	<0.0001	0.0010
Basal MT: Faster	T	T	T	A	C	0.0096	−45.78 (−55.07 to −36.5)	<0.0001	0.0010
Basal MT: Faster	T	T	C	G	T	0.0853	−26.89 (−46.84 to −6.95)	0.0083	0.0553
Basal MT: Faster	T	C	T	G	T	0.0403	−36.03 (−65.98 to −6.09)	0.0180	0.0900
Peak MT: Slower	G	T	C	A	T	0.0071	141.91 (126.42 to 157.39)	<0.0001	0.0010
Peak MT: Slower	G	C	T	G	C	0.0235	66.54 (29.82 to 103.27)	0.0004	0.0026
Peak MT: Faster	T	T	T	A	C	0.0090	−42.88 (−53.57 to −32.19)	<0.0001	0.0010
Difference MT: Lower	G	C	T	G	T	0.0382	−25.16 (−43.17 to −7.15)	0.0062	0.1240

*Associations were sorted by effect, then statistical significance and then intensity of the effect. RT; reaction time; MT, motor time. P-values were adjusted by sex. Pc-values correspond to the corrected P values calculated according to the FDR procedure*.

## Discussion

Genetic variation in *GABR* genes has been related to ethanol effects in animal models, with controversial findings (Hanchar et al., 2004; Wallner et al., 2006; Borghese and Harris, 2007; Botta et al., 2007; Jia et al., 2007; Korpi et al., 2007; Lovinger and Homanics, 2007; Mody et al., 2007; Olsen et al., 2007; Santhakumar et al., 2007). This study analyzes for the first time the effect of common human *GABR* missense polymorphisms in reaction time, motor time and low-dose ethanol effects *in vivo*. Human *GABR* genes showed a modest inter-individual variability as compared to other genes. No common missense or stop polymorphisms have been described for *GABRA*1, 2, 3, and 5. In contrast, the genes *GABRA4* and *GABRA6* have one common missense polymorphism each. These genes encode subunits that have been reported to be related to low-dose ethanol effects (Hanchar et al., 2004; Wallner et al., 2006; Lovinger and Homanics, 2007) and, indeed, one of these polymorphisms is associated in this study to inter-individual differences in reaction times and in ethanol effects (*GABRA4* rs2229940), whereas the other one is related to inter-individual differences in the motor times (*GABRA6* rs4454083) although not to ethanol effects. In both cases, variant subunits are associated with faster times, which is consistent with a functional impairment in inhibitory receptors, such as GABA-A receptors. The amino acid substitution caused by the *GABRA4* polymorphism rs2229940 is predicted as deleterious by using SIFT and as benign by using PolyPhen, and it was reported to display a marginal association with nicotine dependence (Agrawal et al., 2008, 2009). Predictions for the *GABRA6* polymorphism are also deleterious with SIFT, with low confidence, possibly damaging with PolyPhen, but no clinical associations have been yet described.

Besides GABRα4 and GABRα6, the GABA-A subunit combinations containing GABRβ3 and GABRδ are in the model proposed by Hanchar et al. as the primary targets for ethanol (Hanchar et al., 2004). *GABRB3* has two common missense polymorphisms (Table 1) that are at complete linkage, and that in the present study are not associated to reaction times, motor times or ethanol effects (Tables 3, 4). As for *GABRD*, no common missense SNPs have been described (Table 1). Hence, among ethanol-sensitive subunit combinations, the strongest candidates to play a role in the genetic variability of ethanol effects are GABRα4 and GABRα6.

The rest of the common missense *GABR* gene variations did not show in this study a major association with reaction times, motor times, or ethanol effects (Tables 3, 4). Regarding the putative clinical associations for these polymorphisms, the *GABRG2* SNP designated as rs211035 was previously investigated in relation to epilepsy with negative results (Dixit et al., 2016). It has been proposed that the *GABR*E SNP designated as rs1139916 affects the binding of extracellular ligands, and it has been associated with migraine risk in women, with carriers of variant alleles being at decreased risk (Quintas et al., 2013). Our own findings confirm this observation (García-Martín et al., 2018). In addition, a weak association of this SNP with some types of breast cancer has been identified in an exome sequencing study (Li et al., 2015). The *GABRP* rs1063310 SNP has been analyzed as a putative predisposing factor for autism, although no significant association was identified (Ma et al., 2005). Association of the *GABRQ* SNP rs3810651 with response to antidepressants has been claimed in a single study carried out on Chinese individuals (Pu et al., 2013). This SNP has also been analyzed as a putative risk factor for essential tremor, with negative findings (Garcia-Martin et al., 2011a). A putative association of heterozygosity (T/A genotype) with increased risk of developing migraine has been described (Quintas et al., 2013), although these findings could not be replicated by our group (García-Martín et al., 2018). *GABRR1* SNPs rs1186902 and rs12200969 were analyzed as putative risk factors related to ethanol dependence, but no association was identified (Xuei et al., 2010). Conversely, the *GABRR2* SNP rs282129 was significantly related to ethanol dependence (Xuei et al., 2010), and recently it has been claimed to be related to cognitive ability (Ma et al., 2017).

In summary, we identified for the first time functional consequences *in vivo* of two common missense *GABR* polymorphisms. These concern the subunits GABRα4 and GABRα6, which have been reported to be part of sensitive subunit combinations to ethanol. It could be speculated that GABA-A receptors containing such subunits could possibly cause selective changes in reaction and motor time, respectively, by being expressed in different brain regions. Interesting findings regarding a differential effect of extrasynaptic and synaptic GABA inhibition with similar GABA concentrations have been reported (Mooney et al., 2017), and these findings raise evidences supporting effects related to receptor location. The findings regarding the effect of these polymorphisms on reaction times and movement times may have additional clinical implications in movement disorders: Patients with essential tremor and Parkinson's disease have slower reaction times and/or motor times as compared to healthy subjects (Montgomery et al., 2000; Jimenez-Jimenez et al., 2010). In patients with restless syndrome motor impairment exists too, although it is restricted to some movements (Jimenez-Jimenez et al., 2009). GABR alteration may be related to the clinical presentation of movement disorders: for instance, increased expression of GABRα4 has been described in tremor rats (Mao et al., 2011), and our own findings suggest that the *GABRA4* rs2229940 T/T genotype is related to younger age at onset of restless legs syndrome (Jiménez-Jiménez et al. submitted). In the light of the findings obtained in the present study, it would be interesting to verify in further studies whether the *GABRA4* rs2229940 and the *GABRA6* rs4454083 polymorphisms, isolated or combined, are related to clinical presentation, and particularly to severity, reaction time or motor impairment, in patients suffering from movement disorders.

## Author contributions

EG-M, JA: conception and design of the work, interpretation of data, drafting the work, final approval of the version to be published and agreement to be accountable for all aspects of the work in ensuring that questions related to the accuracy or integrity of any part of the work are appropriately investigated and resolved. MR, SG, LR-S: Data acquisition and analysis, interpretation of data for the work, revising critically the work for important intellectual content, final approval of the version to be published and agreement to be accountable for all aspects of the work in ensuring that questions related to the accuracy or integrity of any part of the work are appropriately investigated and resolved. JC-G, JP, HA-N, FJ-J: Interpretation of data for the work, revising critically the work for important intellectual content, final approval of the version to be published and agreement to be accountable for all aspects of the work in ensuring that questions related to the accuracy or integrity of any part of the work are appropriately investigated and resolved.

### Conflict of interest statement

The authors declare that the research was conducted in the absence of any commercial or financial relationships that could be construed as a potential conflict of interest.

## Footnotes

^1^http://phase3browser.1000genomes.org/Homo_sapiens/Info/Index.

^2^http://phase3browser.1000genomes.org/Homo_sapiens/Info/Index.
